# A semi-supervised learning approach for automated 3D cephalometric landmark identification using computed tomography

**DOI:** 10.1371/journal.pone.0275114

**Published:** 2022-09-28

**Authors:** Hye Sun Yun, Chang Min Hyun, Seong Hyeon Baek, Sang-Hwy Lee, Jin Keun Seo

**Affiliations:** 1 School of Mathematics and Computing (Computational Science and Engineering), Yonsei University, Seoul, South Korea; 2 Department of Oral and Maxillofacial Surgery, Oral Science Research Center, College of Dentistry, Yonsei University, Seoul, South Korea; Universiti Malaysia Pahang, MALAYSIA

## Abstract

Identification of 3D cephalometric landmarks that serve as proxy to the shape of human skull is the fundamental step in cephalometric analysis. Since manual landmarking from 3D computed tomography (CT) images is a cumbersome task even for the trained experts, automatic 3D landmark detection system is in a great need. Recently, automatic landmarking of 2D cephalograms using deep learning (DL) has achieved great success, but 3D landmarking for more than 80 landmarks has not yet reached a satisfactory level, because of the factors hindering machine learning such as the high dimensionality of the input data and limited amount of training data due to the ethical restrictions on the use of medical data. This paper presents a semi-supervised DL method for 3D landmarking that takes advantage of anonymized landmark dataset with paired CT data being removed. The proposed method first detects a small number of easy-to-find reference landmarks, then uses them to provide a rough estimation of the all landmarks by utilizing the low dimensional representation learned by variational autoencoder (VAE). The anonymized landmark dataset is used for training the VAE. Finally, coarse-to-fine detection is applied to the small bounding box provided by rough estimation, using separate strategies suitable for the mandible and the cranium. For mandibular landmarks, patch-based 3D CNN is applied to the segmented image of the mandible (separated from the maxilla), in order to capture 3D morphological features of mandible associated with the landmarks. We detect 6 landmarks around the condyle all at once rather than one by one, because they are closely related to each other. For cranial landmarks, we again use the VAE-based latent representation for more accurate annotation. In our experiment, the proposed method achieved a mean detection error of 2.88 mm for 90 landmarks using only 15 paired training data.

## 1 Introduction

Cephalometric analysis is commonly used by dentists, orthodontists, and oral and maxillofacial surgeons to provide morphometrical guidelines for diagnosis, surgical planning, growth analysis, and treatment planning by analyzing dental and skeletal relationships in the craniofacial complex [1]. It is based on cephalometric landmarks, which serve as proxy to the skull morphological data pertaining to craniofacial characteristics [2]. Conventional cephalometric analysis uses two-dimensional (2D) cephalometric radiographs (lateral and frontal radiographs), which have drawbacks including geometric distortions, superimpositions, and the dependence on correct head positioning [3]. Due to recent advances in image processing techniques and the need for accurate craniofacial analysis, a three-dimensional (3D) approach to the cephalometric landmarks obtaining 3D computerized tomography (CT) images is gaining preference over the conventional 2D techniques [4–6].

Recently, there have been many studies conducted on automated cephalometric landmark identification that aims to find the landmarks and enable immediate cephalometric analysis, because manual landmarking and cephalometric analysis are labor-intensive and cumbersome tasks even for the trained experts. Due to recent advances in deep learning techniques, the automated annotation of 2D cephalometric landmarks may now be used for clinical application [7, 8]. Conversely, automated 3D cephalometric tracing (for 90 landmarks) may not yet be utilized in clinical applications, wherein the required average error is commonly designated to be less than 2 mm [9–13]. The high dimensionality of the input data (e.g., 512 × 512 × 512) and limited number of training data are the main factors that hinder the training of deep learning networks for learning the 3D landmark positional vectors from 3D CT data. Moreover, due to the current legal and ethical restrictions on medical data, it is very difficult to utilize CT data from patients.

To overcome the above-mentioned learning problems caused by the high input dimensions and training data deficiencies, the method proposed in this study utilizes semi-supervised learning that takes advantage of a large number of anonymized landmark dataset (without using the corresponding CT dataset) which have been used in surgical planning and treatment evaluation. We use these landmark dataset to obtain their low dimensional representations, reducing the dimensions of the total landmark vectors (270 = 90 × 3 dimension) to only 9 latent variables via a variational autoencoder (VAE) [14]. For training the VAE, a normalized landmark dataset is used to efficiently learn skull shape variations while ignoring unnecessary scaling factors. With this dimensionality reduction technique, the positions of all 90 landmarks can be roughly estimated by identifying a small number of easy-to-find reference landmarks (10 landmarks), which can be accurately and reliably identified via a simple deep learning method [11].

The rough estimation of all landmarks is used to provide a small 3D bounding box for each landmark in the 3D CT images. Following this, we apply convolutional neural networks (CNNs) to these small bounding boxes to enable the accurate placement of landmarks. Our fine detection strategy is divided into two parts; mandible and cranium. It is desirable to accurately capture the morphological variability of the mandible because the shape of the mandible can be affected by a variety of factors, including the masticatory occlusal force, muscular force, functional activity such as breathing and swallowing, and age [15]. Noting that landmarks on the mandible represent morphological features of a 3D mandibular surface geometry, we apply 3D CNN to a segmented image of the mandible (separated from the cranium). We follow a recent study [16] for a segmentation method to separate the mandible from the cranium.

Because several landmarks around the condyle are closely related to each other, it is better to detect these landmarks all at once. For the landmarks on the midsagittal plane, it is better to further reduce the dimensionality of the input by using a partially integrated 2D image of the midsagittal plane. For the remaining landmarks lying on the cranium, we again use the anonymized landmark dataset to obtain a more accurate latent representation of all landmarks on the cranium, due to its rigidity. The proposed approach achieved a mean detection error of 2.88 mm for 90 landmarks, which nearly meets the clinically acceptable precision standard. It should be emphasized that this accuracy has been achieved using a very small amount of training data.

## 2 Method

We begin by introducing the following notations. Five easy-to-find reference landmarks (CFM, Bregma, Na, and Po (L/R)) are used as the basis for constructing a coordinate system to determine the midsagittal and axial planes, and they are utilized for data normalization (methods for obtaining these five reference landmarks will be described in Section 2.1).

- ***x*** denotes a 3D CT image, which is defined on a voxel grid Ω ≔ {*v* = (*v*_1_, *v*_2_, *v*_3_):*v*_*j*_ = 1, ⋯, 512 for *j* = 1, 2, 3}. Here, we set *v*_1_ as the normal direction of the midsagittal plane.
- ***x***_*b*_ denotes a binarized CT image of ***x*** (i.e., skull segmentation from the CT image), defined by
  $$\begin{array}{c} x_{b}=\{\begin{array}{cc} x_{b}\left(v_{1},v_{2},v_{3}\right)=1 & \;\text{if}\;x(v_{1},v_{2},v_{3})\geq \rho \\ x_{b}\left(v_{1},v_{2},v_{3}\right)=0 & \;\text{otherwise}\; \end{array} \end{array}$$ (1)
  where *ρ* is a thresholding value. In our experiment, the value of *ρ* was consistently chosen as *ρ* = 500*HU*, which is known as an effective choice for thresholding-based bone segmentation [17].
- ***x***^mid^ denotes a partially integrated 2D image of ***x***_*b*_ in the normal direction of the midsagittal plane, defined by
  $$\begin{array}{c} x^{\text{mid}}=\sum_{v_{1}=a}^{b}x_{b}\left(v_{1},v_{2},v_{3}\right) \end{array}$$ (2)
  where [*a*, *b*] determines the truncated volume of ***x***_*b*_.
-
$R^{\text{cr}}\in R^{138(=46\times 3)}$ and $R^{\text{md}}\in R^{132(=44\times 3)}$ denote the concatenated vectors of 46 cranial and 44 mandibular 3D landmarks, respectively. The entirety of the landmarks $R\in R^{270(=90\times 3)}$ is defined by $R≔[R^{\text{cr}},R^{\text{md}}]$. See S1 Table for more detailed information of the landmarks.
-
$R_{♯}^{\text{cr}}\in R^{24(=8\times 3)}$ denotes a concatenated vector of the landmarks (Bregma, CFM, Na, ANS, Or (L/R), and Po (L/R)) in the cranium and $R_{♯}^{\text{md}}\in R^{6(=2\times 3)}$ denotes a concatenated vector of the landmarks (MF (L/R)) in the mandible. A reference landmark vector $R_{♯}\in R^{30(=10\times 3)}$ is defined by $R_{♯}=\left[R_{♯}^{\text{cr}},R_{♯}^{\text{md}}\right]$.

**Fig 1:**
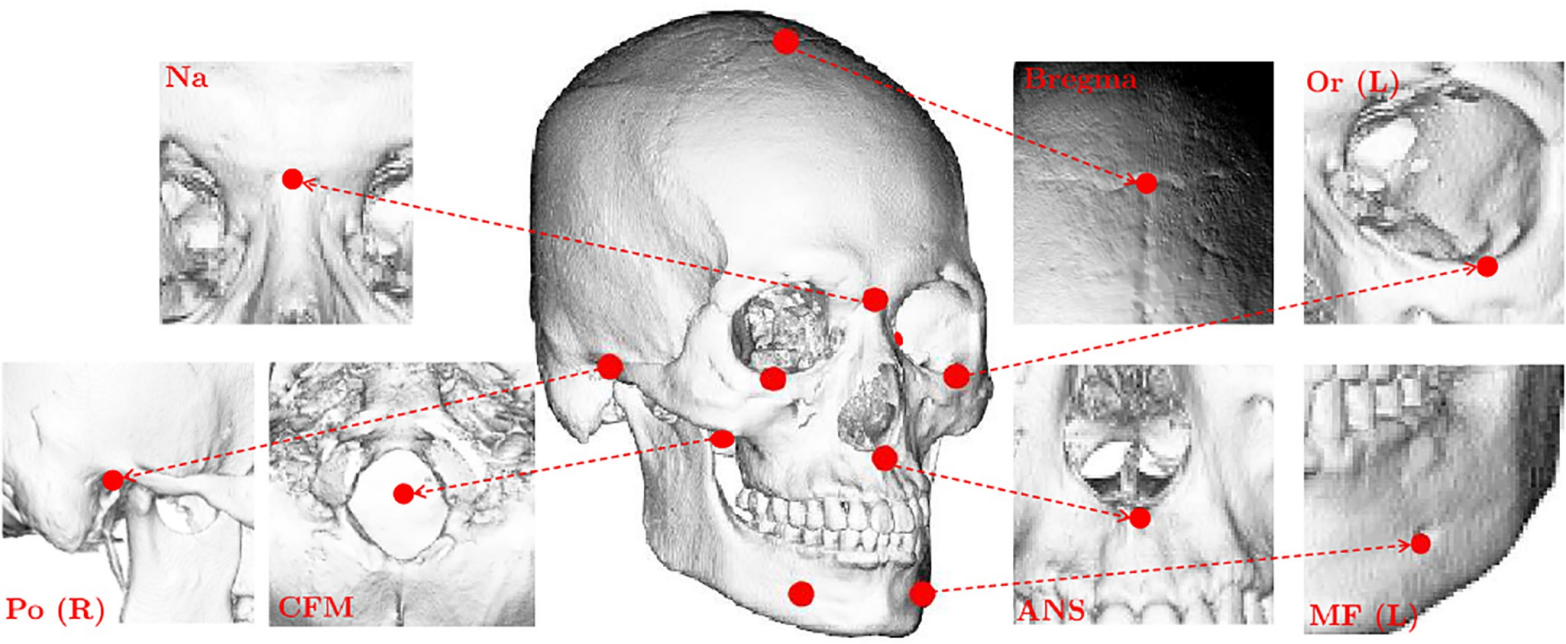
Reference landmarks. These are easy-to-find through CNN with input of the illuminated images because they have strong geometric cues that can be revealed in illuminated 2D images.

We mention here details of reference landmarks; Bregma is the point of junction of the coronal and sagittal sutures of the skull. (i) CFM, (ii) Na, (iii) ANS, (iv) Or, (v) Po, and (vi) MF are the abbrevations of (i) the center of foramen magnum, (ii) nasion, (iii) anterior nasal spine, (iv) orbitale, (v) porion, and (vi) mental foramen, which are defined by (i) the center of an opening for spinal cord, (ii) the center of the midline bony depression between the eyes, where the frontal and two nasal bones meet, just below the glabella, (iii) the projection formed by the fusion of the two maxillary bones at the intermaxillary suture, (iv) lower most point on the lower margin of the left or right orbit, (v) the most superior point of the upper margin of each ear canal, and (vi) a bilateral opening in the vestibular portion of the mandible through which nerve endings, such as the mental nerve, emerge. See Fig 1.

The 3D cephalometric landmarking aims to develop a function f:x↦R that maps a 3D CT image ***x*** to all landmarks R. To learn the landmark detection map *f*, deep learning techniques can be used. Unfortunately, due to the legal and ethical restrictions on medical data, a few paired data are available. This severe shortage of paired data makes it difficult to obtain an accurate and reliable map f:x↦R in the following supervised learning framework:

$$\begin{array}{c} f=\underset{f\in N\text{et}}{\text{argmin}}\frac{1}{N_{p}}\sum_{i=1}^{N_{p}}{\left\|f\left(x^{\left(i\right)}\right)-R^{\left(i\right)}\right\|}_{2}^{2}, \end{array}$$ (3)

where *N*_*p*_ is the small number of paired training data, $\left\{\left(x^{\left(i\right)},R^{\left(i\right)}\right):i=1,\cdots ,N_{p}\right\}$ is a paired dataset, Net is a deep learning network, and ‖ ⋅ ‖ is the standard Euclidean norm. In our study, only 15 paired data are available (i.e., *N*_*p*_ = 15). Even with a certain amount of paired data, the learning process (3) of the direct detection map *f* can be difficult because the dimension of the input image is very large (greater than 10^8^).

The proposed method attempts to address this problem by taking advantage of a semi-supervised learning framework that permits the utilization of the *N*_*l*_ number of anonymized landmark data ${\left\{R^{\left(N_{p}+i\right)}\right\}}_{i=1}^{N_{l}}$ whose corresponding CT data are not provided. In this research, specifically, 229 anonymized data (i.e., *N*_*l*_ = 229) are utilized.

**Fig 2:**
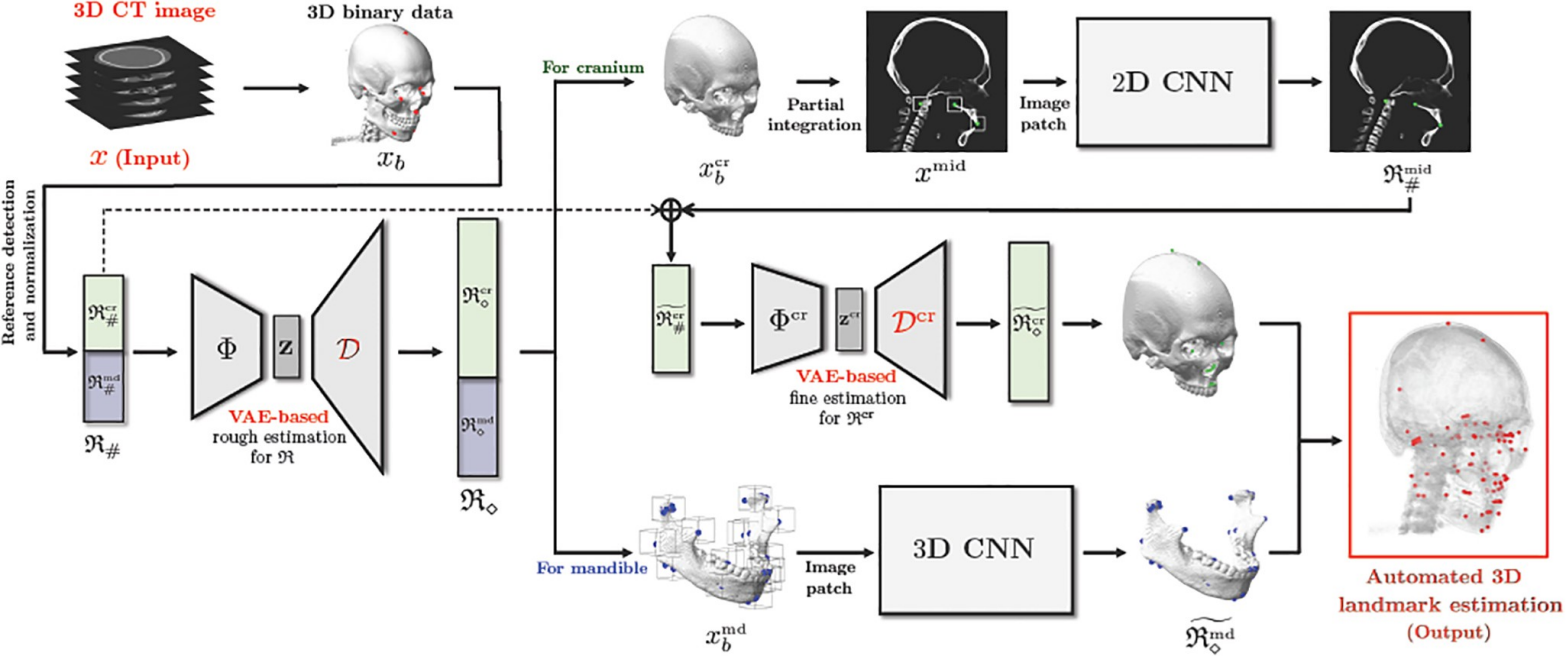
Schematic diagram of the proposed method for the 3D landmark annotation system.

As shown in Fig 2, the proposed method comprises the following three main steps: (i) To obtain easy-to-find reference landmarks $R_{♯}$, we apply CNN with 2D illuminated images generated from a binarized CT image ***x***_*b*_ and normalize the output with respect to the cranial volume. (ii) A rough estimation of entire landmarks R is obtained using the partial knowledge $R_{♯}$ and a VAE-based low dimensional representation of R. (iii) Using this estimation, coarse-to-fine detection for R is conducted, wherein separate strategies are utilized for the mandibular and cranial landmarks. For the mandibular landmarks, the landmarks are accurately identified by applying 3D patch-based CNNs to capture the morphological features on a 3D surface geometry associated with the landmarks, wherein an input patch is extracted based on the coarse estimation. For cranial landmarks, we first detect three landmarks lying on the midsagittal plane by applying a 2D CNN whose input is an extracted 2D patch from a partially integrated image ***x***^mid^ in basis of the coarse estimation. By utilizing the three finely-detected landmarks and cranial reference landmarks $R_{♯}^{\text{cr}}$ as the partial information of $R^{\text{cr}}$, the remaining cranial landmarks are finely annotated via a VAE-based local-to-global estimation utilizing the same method in the previous step.

Each of these steps is described in detail as follows.

### 2.1 Detection of easy-to-find reference landmarks and uniform scaling for skull normalization with respect to the cranial volume

The first step of the proposed method is to find 10 reference landmarks $R_{♯}$ from a given ***x***. Initially, a CT image ***x*** is converted into a binarized image ***x***_*b*_ by (1). From ***x***_*b*_, 2D illuminated images are generated by manipulating various lighting and viewing directions (see Fig 1). By applying VGGNet [18] to these illuminated images, the reference landmarks $R_{♯}$ are accurately and automatically identified. This detection method is based on that presented in the recent study [11].

Using these identified reference landmarks, data normalization is conducted for efficient feature learning of skull shape variations in further steps. By applying a uniform scaling with respect to the cranial volume, the landmark vector $R_{♯}$ is normalized, wherein the cranial volume is defined via a product of the distance between the *v*_1_-coordinate of Po (L) and Po (R) (cranial length), the distance between the *v*_2_-coordinate of Po (L) and Na (depth), and the distance between the *v*_3_-coordinate of CFM and Bregma (height). This data normalization minimizes the positional dependencies of the landmarks on the translation, rotation, and overall size of the skull; therefore, shape information of the skull (regarding facial deformities) can be effectively learned in further VAE-based steps. From here on, we will denote all landmark vectors as normalized vectors (e.g., R and $R_{♯}$ are normalized vectors for total landmarks and reference landmarks).

### 2.2 Rough estimation of all landmarks from reference landmarks using VAE-based low dimensional representation

This subsection provides a method for roughly estimating all landmarks R from the reference landmarks $R_{♯}$ that are accurately annotated in the previous step. Based on the method in [13], we build a bridge that connects $R_{♯}$ and R by taking advantage of a low-dimensional representation of R learned by the variational autoencoder (VAE) [14].

**Fig 3:**
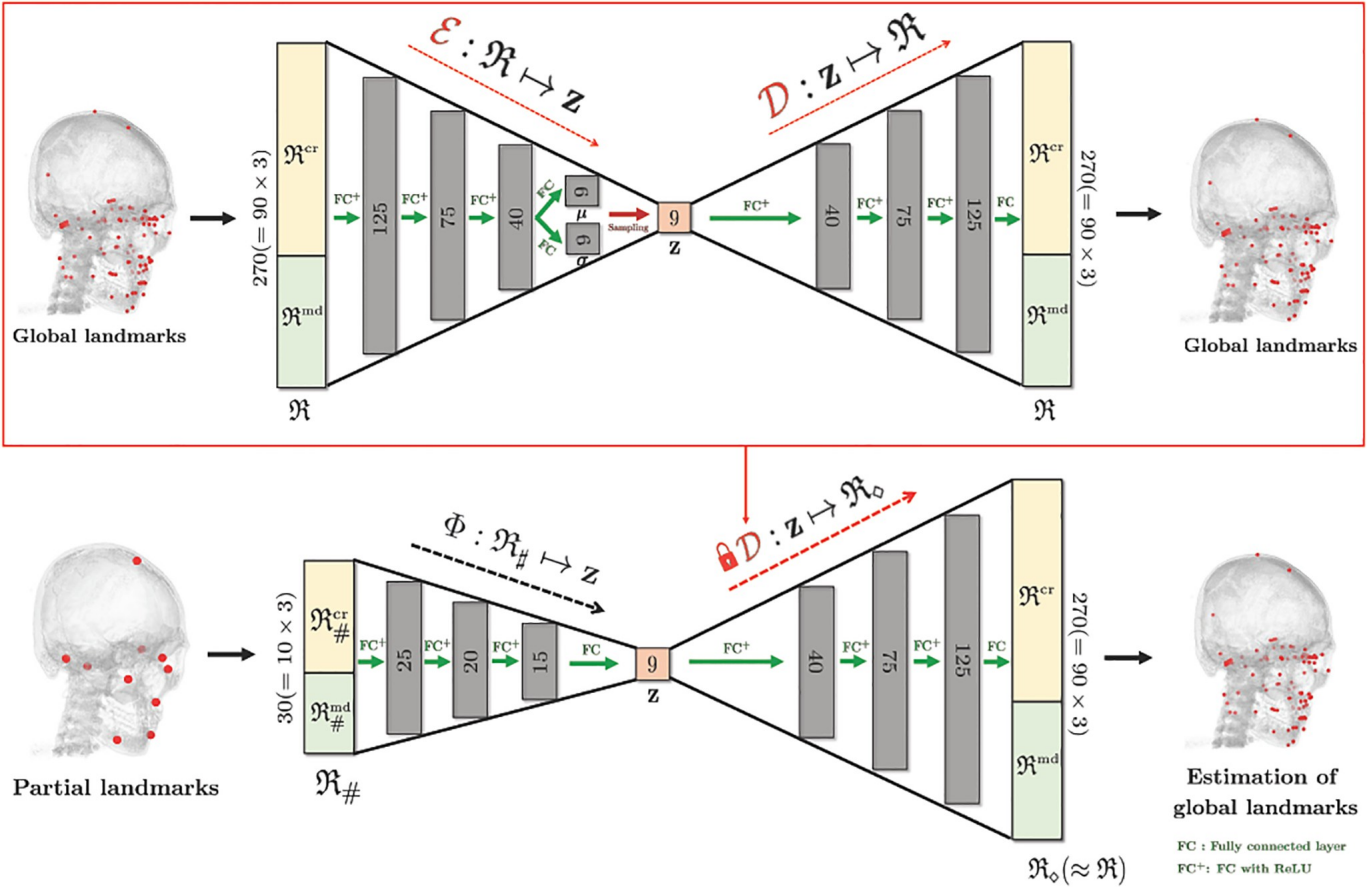
Initial estimation of all 90 landmarks R using the knowledge of 10 reference landmarks $R_{♯}$. This is possible because all landmarks R can be roughly represented by only 9 latent variables.

The rough estimation obtained from $R_{♯}$, denoted by $R_{◇}$, is given by

$$\begin{array}{c} R_{◇}=D\circ \Phi \left(R_{♯}\right) \end{array}$$ (4)

where $D\circ \Phi :R_{♯}\mapsto R_{◇}$ is a local-to-global landmark estimation map as described in Fig 3. The map is constructed as follows: First, we train VAE that consists of an encoder (E) and a decoder (D), to learn low dimensional representation of R. Afterwards, we train the nonlinear map Φ that provides $\Phi \left(R_{♯}\right)\approx E\left(R\right)$ so that $D\circ \Phi (R_{♯})\approx R$.

Specifically, the VAE learns an encoder E:R↦z and a decoder D:z↦R, where $z\in R^{d}$ is a *d*-dimensional latent variable (*d* ≪ 270). The maps E and D learn landmark patterns by leveraging dataset ${\left\{R^{\left(i\right)}\right\}}_{i=1}^{N_{t}}$ that consists of the unpaired landmark dataset as well as the paired dataset. The training is achieved via the following energy minimization sense:

$$\begin{array}{c} \left(E,D\right)=\underset{\left(E,D\right)\in VAE}{\text{argmin}}\overset{N_{t}}{\underset{i=1}{\sum }}(\|D\circ E\left({ℜ}^{\left(i\right)}\right)-{ℜ}^{\left(i\right)}{\|}_{2}^{2}+D_{KL}\left(N\left(\mu^{\left(i\right)},\Sigma^{\left(i\right)}\right)\|N\left(0,I\right)\right)) \end{array}$$ (5)

where *N*_*t*_ = *N*_*p*_ + *N*_*l*_ is the total number of training landmark data, VAE is a class of functions in the form of a given VAE network, $N(\mu^{\left(i\right)},\Sigma^{\left(i\right)})$ is a *d*-dimensional normal distribution with a mean *μ*^(*i*)^ and a diagonal covariance matrix Σ^(*i*)^ = *diag*((*σ*^(*i*)^(1))^2^, ⋯, (*σ*^(*i*)^(*d*))^2^), N(0,I) is a standard normal distribution, and the last term in the loss function is the Kullback-Leibler (KL) divergence defined by

$$\begin{array}{cc} & D_{KL}\left(N\left(\mu^{\left(i\right)},\Sigma^{\left(i\right)}\right)\|N\left(0,I\right)\right)\\ & =\frac{1}{2}\sum_{l=1}^{d}\left(\mu^{\left(i\right)}{\left(l\right)}^{2}+\sigma^{\left(i\right)}{\left(l\right)}^{2}-\text{log}\sigma^{\left(i\right)}\left(l\right)-1\right) \end{array}$$ (6)

Here, *μ*^(*i*)^ = (*μ*^(*i*)^(1), ⋯, *μ*^(*i*)^(*d*)) and *σ*^(*i*)^ = (*σ*^(*i*)^(1), ⋯, *σ*^(*i*)^(*d*)) are the mean and standard deviation vectors obtained in the interim of the encoding process of an *i*-th training data $R^{\left(i\right)}$ (i.e., $E(R^{\left(i\right)})$).

The encoder E can be expressed in the following nondeterministic form:

$$\begin{array}{c} E\left(R\right)=z≔\mu +\sigma ⊙h_{\text{noise}} \end{array}$$ (7)

where **h**_noise_ is a noise sampled from N(0,I), ⊙ is the Hadamard product (i.e., element-wise product), and vectors *μ* and *σ* are given by

$$\begin{array}{cc} & \mu =E_{4}^{\mu }h,\sigma =E_{4}^{\sigma }h\\ & h=\text{ReLU}(E_{3}\text{ReLU}\left(E_{2}\text{ReLU}\left(E_{1}R\right)\right)) \end{array}$$ (8)

Here, the matrices $\left\{E_{1},E_{2},E_{3},E_{4}^{\mu },E_{4}^{\sigma }\right\}$ represent fully-connected layers and ReLU is an element-wise activation function defined by ReLU(*t*) = max(*t*, 0). The decoder D is the reverse process of the encoder E, which can be represented by

$$\begin{array}{c} D\left(z\right)=D_{1}\text{ReLU}\left(D_{2}\text{ReLU}\left(D_{3}\text{ReLU}\left(D_{4}z\right)\right)\right) \end{array}$$ (9)

where the matrices {**D**_1_, **D**_2_, **D**_3_, **D**_4_} represent fully-connected layers. The detailed network architecture is described in the red box of Fig 3.

After pretraining the VAE, the nonlinear map $\Phi :R_{♯}\to z$ is learned, which connects reference landmarks $R_{♯}$ with a latent variable z=E(R). The architecture of the map Φ is a fully-connected neural network as described in Fig 3. The training is achieved by the following minimization sense:

$$\begin{array}{c} \Phi =\underset{\Phi }{\text{argmin}}\sum_{i}{\left\|\Phi \left(R_{♯}^{\left(i\right)}\right)-z^{\left(i\right)}\right\|}_{2}^{2} \end{array}$$ (10)

Here, we remark that Φ relies on the pretrained VAE, whose encoder (E) is used only for training and decoder (D) is the component of the local-to-global detection map.

The resultant map D∘Φ estimates all landmarks R from the partial knowledge $R_{♯}$, based on the learned patterns of landmarks by VAE.

### 2.3 Coarse-to-fine detection

This subsection explains coarse-to-fine detection obtained using the initial estimation $R_{◇}$. We put a final touch on $R_{◇}$ by utilizing CT image data. The coarse-to-fine detection is based on suitable strategies that rely on the landmark locations (i.e. on the mandible or cranium). The details are explained in the following subsections.

#### 2.3.1 Mandible-cranium segmentation

In the binarized skull image ***x***_*b*_, we segment the mandible and cranium separately using the connected component labeling (CCL) technique [19]. Among all connected components generated from the CCL method, the largest component and the second largest are the cranium and the mandible respectively. The segmented cranium and mandible images are denoted as $x_{b}^{\text{cr}}$ and $x_{b}^{\text{md}}$ (as shown in Fig 2). Using these images and the rough estimation $R_{◇}$, the following fine detection processes are conducted.

#### 2.3.2 Detection of mandibular landmarks

For the landmarks on the mandible being articulated to the skull, a patch-based 3D CNN is applied to capture the morphological variability of the 3D mandibular surface geometry associated with the landmarks.

Let $R_{◇}^{j}\in R^{3}$ be a roughly estimated position of a landmark with index *j* in $R_{◇}$. See S1 Table for the details of the landmark index. For each mandibular landmark (i.e., *j* ∈ {49, ⋯, 90}), we extract a 3D image patch ${\left(x_{b}^{\text{md}}\right)}_{\left(\eta ,R_{◇}^{j}\right)}$ that is defined by a cube with edge length of *η* and center of $R_{◇}^{j}$. By using 3D CNN, we obtain a map $f_{j}^{\text{md}}:{\left(x_{b}^{\text{md}}\right)}_{\left(\eta ,R_{◇}^{j}\right)}\mapsto {\tilde{R_{◇}}}^{j}$, where ${\tilde{R_{◇}}}^{j}$ is an accurate positional estimation for the landmark with index *j* (i.e., ${\tilde{R_{◇}}}^{j}\approx R^{j}$).

To learn the fine detection map $f_{j}^{\text{md}}$, we generate training dataset by using the paired dataset ${\left\{\left({\left(x_{b}^{\text{md}}\right)}^{\left(i\right)},R^{\left(i\right)}\right)\right\}}_{i=1}^{N_{p}}$: For a given landmark index *j*, we generate

$$\begin{array}{c} \left\{\left({\left(x_{b}^{\text{md}}\right)}_{\left(\eta ,{\left(R_{◇}^{\left(i\right)}\right)}^{j}\right)}^{\left(i\right)},{\left(R^{\left(i\right)}\right)}^{j}\right):i=1,\cdots ,N_{p}\right\} \end{array}$$ (11)

The 3D CNN is trained by the dataset in the following sense:

$$\begin{array}{c} f_{j}^{\text{md}}=\underset{f_{j}^{\text{md}}}{\text{argmin}}\;\;\sum_{i}\;{\left\|f_{j}^{\text{md}}\left({\left(x_{b}^{\text{md}}\right)}_{\left(\eta ,{\left(R_{◇}^{\left(i\right)}\right)}^{j}\right)}^{\left(i\right)}\right)-{\left(R^{\left(i\right)}\right)}^{j}\right\|}_{\ell^{2}}^{2} \end{array}$$ (12)

**Fig 4:**
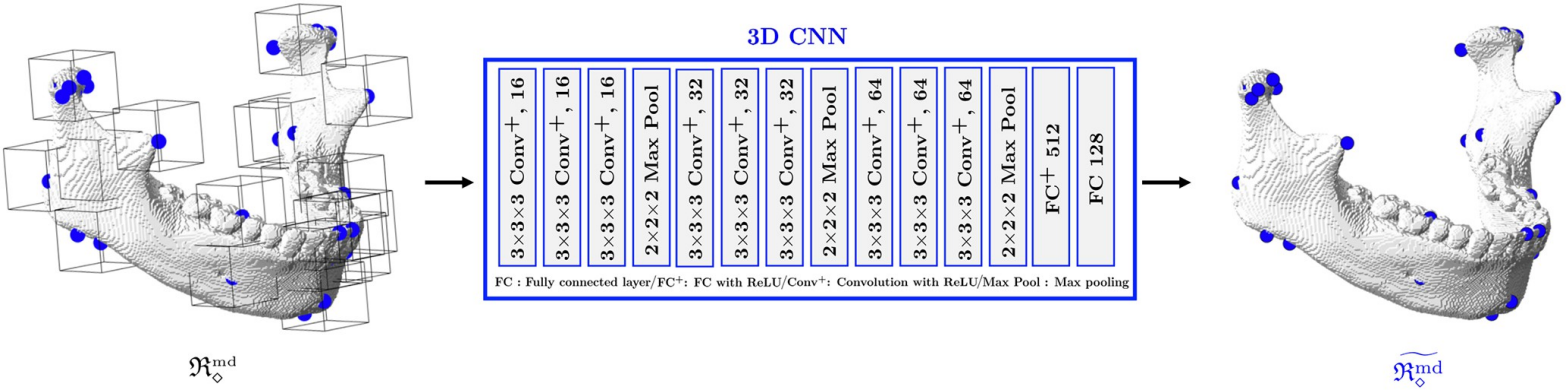
Mandibular landmarks detection. Patch-based 3D CNN is applied to the segmented image of the mandible (separated from the maxilla), in order to capture 3D morphological features of mandible associated with the landmarks. For six landmarks on condyle, we detect them all at once, instead of one by one, because they are positionally related to each other.

In practice, data augmentation using translation and horizontal flip is applied. The architecture of the 3D CNN is a modified version of VGGNet [18], which is described in Fig 4.

In practice, several landmarks are identified in a group at once. We simultaneously identify six landmarks on the condyle (COR, MCP, LCP, Cp, Ct-in, and Ct-out) that are positionally related to one another, as well as landmarks with bilaterality (e.g. left/right mandibular foramen) that are associated with the symmetric structure of the mandible. For this group detection, we construct a 3D CNN to produce a concatenated vector of all landmark positions on the same group from one 3D image patch. Here, COR is the abbreviation of coronoid point, defined by the anterior process of the superior border of the ramus of the mandible. (i) MCP, (ii) LCP, (iii) Cp, (iv) Ct-in, and (v) Ct-out are the abbreviations of (i) medial condylar point, (ii) lateral condylar point, (iii) posterior condylar point, (iv) medial temporal condylar point, and (v) lateral temporal condylar point.

#### 2.3.3 Detection of cranial landmarks

**Fig 5:**
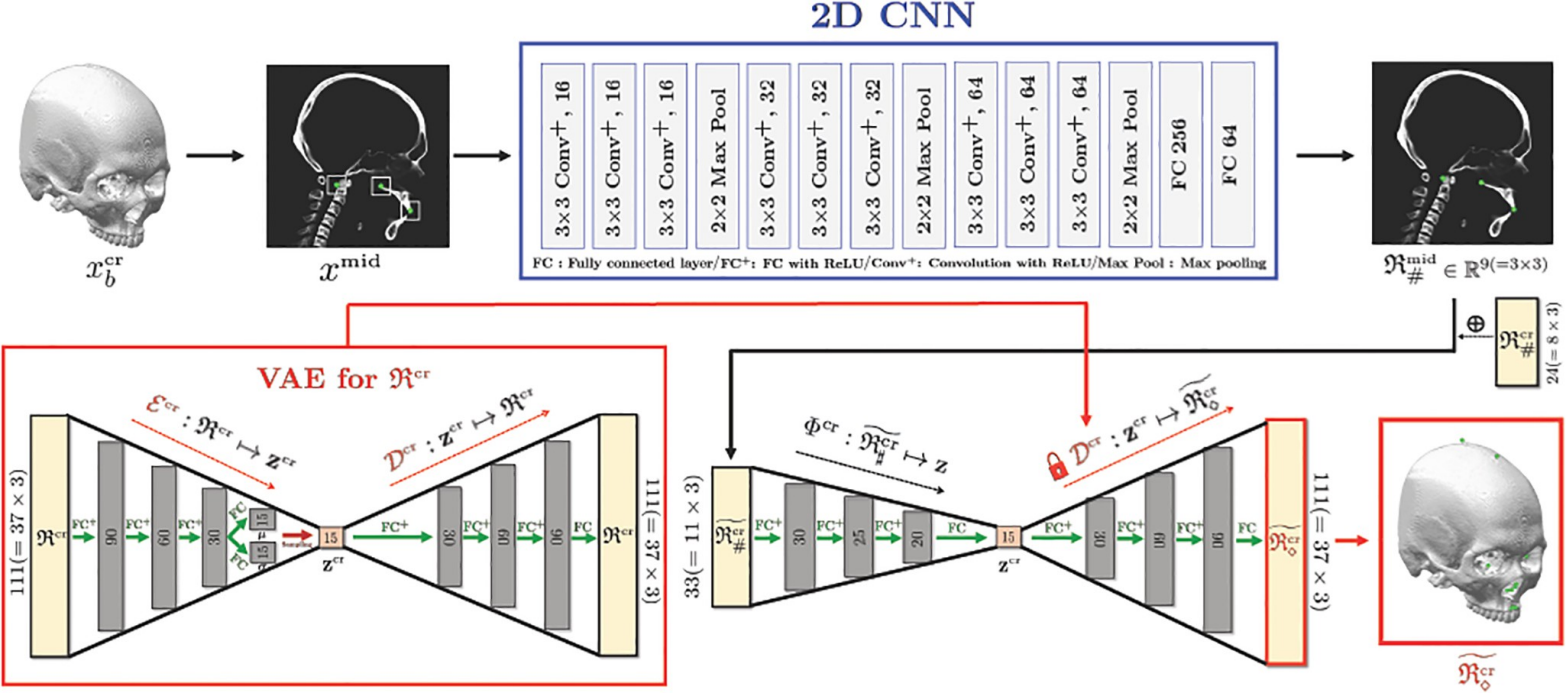
3D cranial landmark detection using VAE-based low dimensional representation combined with easy-to-find landmarks. Here, the entire cranial landmarks $R^{\text{cr}}$ are estimated directly from the knowledge of the reference landmarks $R_{♯}^{\text{cr}}$ and three landmarks $R_{♯}^{\text{mid}}$ on midsagittal plane that are obtained by 2D CNN.

Landmarks on the cranium that demonstrates rigidity have less variability between subjects. According to [13], cranial landmarks have smaller variance compared to mandibular landmarks with the normalization presented in Section 2.1. Moreover, our empirical experiment shown in Fig 7 demonstrates that the rough local-to-global estimation achieved using the VAE-based low dimensional representation provides more accurate annotations for cranial landmarks. Therefore, we again utilize a VAE-based low dimensional representation in the same manner as in Section 2.2 by using only the cranial landmarks $R^{\text{cr}}$. To increase the detection accuracy, we enrich the partial knowledge of $R^{\text{cr}}$ by accurately detecting three additional cranial landmarks lying near the midsagittal plane (MxDML, Od, and PNS). Here, (i) MxDML, (ii) Od, (iii) PNS are the abbrevation of (i) maxillary dental midline, (ii) odontoid process, and (iii) posterior nasal spine, which are defined by (i) the midsagittal line and point of the maxillary central incisors, usually defined by the junctional line and point of right and left incisal edge and medial surface on maxillary central incisors, (ii) a protuberance (process or projection) of the axis (second cervical vertebra), and (iii) a part of the horizontal plate of the palatine bone of the skull. The overall process is illustrated in Fig 5.

First, we compute a partially integrated image ***x***^mid^ from $x_{b}^{\text{cr}}$ using (2) so that the center of the truncated volume of $x_{b}^{\text{cr}}$ lies on the midsagittal plane. Next, a 2D patch ${\left(x^{\text{mid}}\right)}_{\left(\eta ,R_{◇}^{j}{|}_{v_{2},v_{3}}\right)}$ is extracted, which is defined by a square with edge length of *η* and center of $R_{◇}^{j}{|}_{v_{2},v_{3}}$. Here, $R_{◇}^{j}{|}_{v_{2},v_{3}}$ is a vector eliminating the *v*_1_ component in the $R_{◇}^{j}$ and *j* ∈ {24, 25, 26}. Using a 2D CNN, we learn a function $f_{j}^{\text{cr}}$ that infers an accurate position of a landmark $R^{j}$ in *v*_2_- and *v*_3_-coordinates ($R^{j}{|}_{v_{2},v_{3}}$) from the 2D image patch ${\left(x^{\text{mid}}\right)}_{\left(\eta ,R_{◇}^{j}{|}_{v_{2},v_{3}}\right)}$. The landmark position in the *v*_1_-coordinate is determined by the location of the midsagittal plane.

In the similar manner as in (11), the following training dataset is generated:

$$\begin{array}{c} \left\{\left({\left(x^{\text{mid}}\right)}_{\left(\eta ,{\left(R_{◇}^{\left(i\right)}\right)}^{j}{|}_{v_{2},v_{3}}\right)}^{\left(i\right)},{\left(R^{\left(i\right)}\right)}^{j}{|}_{v_{2},v_{3}}\right):i=1,\cdots ,N_{p}\right\} \end{array}$$ (13)

With the training dataset, the 2D CNN is trained as follows:

$$\begin{array}{c} f_{j}^{\text{cr}}=\underset{f_{j}^{\text{cr}}}{\text{argmin}}\sum_{i}\|f_{j}^{\text{cr}}\left({\left(x^{\text{mid}}\right)}_{\left(\eta ,{\left(R_{◇}^{\left(i\right)}\right)}^{j}{|}_{v_{2},v_{3}}\right)}^{\left(i\right)}\right)-{\left(R^{\left(i\right)}\right)}^{j}{{|}_{v_{2},v_{3}}\|}_{\ell^{2}}^{2} \end{array}$$ (14)

Here, data augmentation using translation is applied. The architecture of the 2D CNN is modified from VGGNet [18], as illustrated in Fig 4.

Let $R_{♯}^{\text{mid}}$ be a concatenated positional vector with cranial reference landmarks $R_{♯}^{\text{cr}}$ and three finely detected landmarks obtained by $f_{j}^{\text{cr}}$. Using this partial knowledge $R_{♯}^{\text{mid}}$, we find accurate cranial landmark positions $\tilde{R_{◇}^{\text{cr}}}$ via

$$\begin{array}{c} \tilde{R_{◇}^{\text{cr}}}=D^{\text{cr}}\circ \Phi^{\text{cr}}\left(R_{♯}^{\text{mid}}\right) \end{array}$$ (15)

where $\Phi^{\text{cr}}:R_{♯}^{\text{mid}}\mapsto z^{\text{cr}}$ is a nonlinear map and $D^{\text{cr}}:z^{\text{cr}}\mapsto R^{\text{cr}}$ is a decoder of VAE. Here, $z^{\text{cr}}\in R^{d^{\text{cr}}}$ is a $d^{\text{cr}}$-dimensional latent variable given by $z^{\text{cr}}=E\left(R^{\text{cr}}\right)$ and $E^{\text{cr}}:R^{\text{cr}}\mapsto z^{\text{cr}}$ is an encoder of VAE. The maps $\left(E^{\text{cr}},D^{\text{cr}}\right)$ and $\Phi^{\text{cr}}$ are trained in the same manner of the method presented in Section 2.2 using cranial landmarks $R^{\text{cr}}$. The detailed architectures of $\left(E^{\text{cr}},D^{\text{cr}}\right)$ and $\Phi^{\text{cr}}$ are illustrated in Fig 5.

## 3 Result

### 3.1 Dataset and experimental settings

Our experiment used a dataset containing 24 paired data (multi-detector CT images and landmark data) and 229 anonymized landmark data. This dataset was provided by Yonsei University, Seoul, Korea. The paired dataset was obtained from normal Korean adult volunteers (9 males and 15 females; 24.22±2.91 years old) with skeletal class I occlusion and was approved by the local ethics committee of the Dental College Hospital, Yonsei University (IRB number: 2–2009-0026). All informed consents were obtained from each subject. Among the 24 paired data, we used 15 data pairs for training (i.e., *N*_*p*_ = 15) and 9 data pairs for testing. The anonymized landmark dataset with 3D landmark coordinates was acquired in an excel format from 229 anonymized subjects with dentofacial deformities and malocclusions (i.e., *N*_*l*_ = 229). Manual landmarking for both dataset was performed by one of the authors (S.-H. Lee) who is an expert in 3D cephalometry with more than 20 years of experience.

Our deep learning method was implemented with Pytorch [20] in a computer system with 4 GPUs (GeForce RTX 1080 Ti), two Intel(R) Xeon(R) CPU E5–2630 v4, and 128GB DDR4 RAM. In the training process, the Adam optimizer [21] was consistently adopted, which is known as an effective adaptive gradient descent method. In our experiment, optimal values of all learning parameters (epoch and learning rate) were empirically selected via cross validation. For image-based methods, 15-fold cross validation was applied, where 15 paired training data were split into 1-fold for validation and the others for training. For the training of VAE parts, 5-fold cross-validation was applied to 244 training data (229 unpaired and 15 paired ones). Fold values were empirically selected, depending on the amount of available training data.

The nonlinear map Φ in (10) can be trained by pairs of $R_{♯}$ and ***z***, where $R_{♯}$ is an estimated vector of 10 reference landmarks in the first step and ***z*** is a latent variable obtained by z=E(R). Here, E is the encoder of the pretrained VAE and R is the corresponding global landmark. Due to the limited number of CT data, only 15 outputs ${\left\{R_{♯}^{\left(i\right)}\right\}}_{i=1}^{15}$ were provided in the first-step. Hence, an additional dataset ${\left\{R_{♯}^{\left(i\right)},z^{\left(i\right)}\right\}}_{i=16}^{244}$ was generated from the unpaired landmark dataset, where $R_{♯}^{\left(i\right)}$ and ***z***^(*i*)^ are given by $R_{♯}^{\left(i\right)}=S_{\text{ub}}\left(R^{\left(i\right)}\right)$ and $z^{\left(i\right)}=E\left(R^{\left(i\right)}\right)$. Here, $S_{\text{ub}}$ denotes a subsampling operator of 10 reference landmarks. This dataset was used in our implementation. Likewise, the map Φ^*cr*^ in (15) was trained as the same manner.

For quantitative evaluation, we used mean detection error (MDE) computed as follows: Let ${\left\{R_{\text{est}}^{\left(i\right)}\right\}}_{i=1}^{N_{eval}}$ be a set of *N*_*eval*_ landmarks output to be evaluated. The MDE is computed by

$$\begin{array}{c} \text{MDE}=\frac{1}{N_{eval}}\sum_{i=1}^{N_{eval}}\left\|R_{\text{est}}^{\left(i\right)}-R_{\text{label}}^{\left(i\right)}\right\| \end{array}$$ (16)

where $R_{\text{label}}^{\left(i\right)}$ is the corresponding ground truth for $R_{\text{est}}^{\left(i\right)}$ and $\|R_{\text{est}}-R_{\text{label}}\|$ is defined by

$$\begin{array}{c} \|R_{\text{est}}-R_{\text{label}}\|=\frac{1}{N_{lmk}}\sum_{j=1}^{N_{lmk}}\left\|R_{\text{est}}^{j}-R_{\text{label}}^{j}\right\| \end{array}$$ (17)

Here, $R_{\text{est}}^{j}$ is the vector corresponding to *j*-th landmark in $R_{\text{est}}$, *N*_*lmk*_ is the number of landmarks contained in $R_{\text{est}}$ (e.g., *N*_*lmk*_ = 90 for entire global landmarks), and $\|R_{\text{est}}^{j}-R_{\text{label}}^{j}\|$ is given by

$$\begin{array}{c} \|R_{\text{est}}^{j}-R_{\text{label}}^{j}\|=\sqrt{\sum_{k=1}^{3}(R_{\text{est}}^{j}{|}_{v_{k}}-R_{\text{label}}^{j}{{|}_{v_{k}})}^{2}} \end{array}$$ (18)

where $R^{j}{|}_{v_{k}}$ denotes the *v*_*i*_-coordinate of $R^{j}$.

### 3.2 Results of reference landmark detection

**Fig 6:**
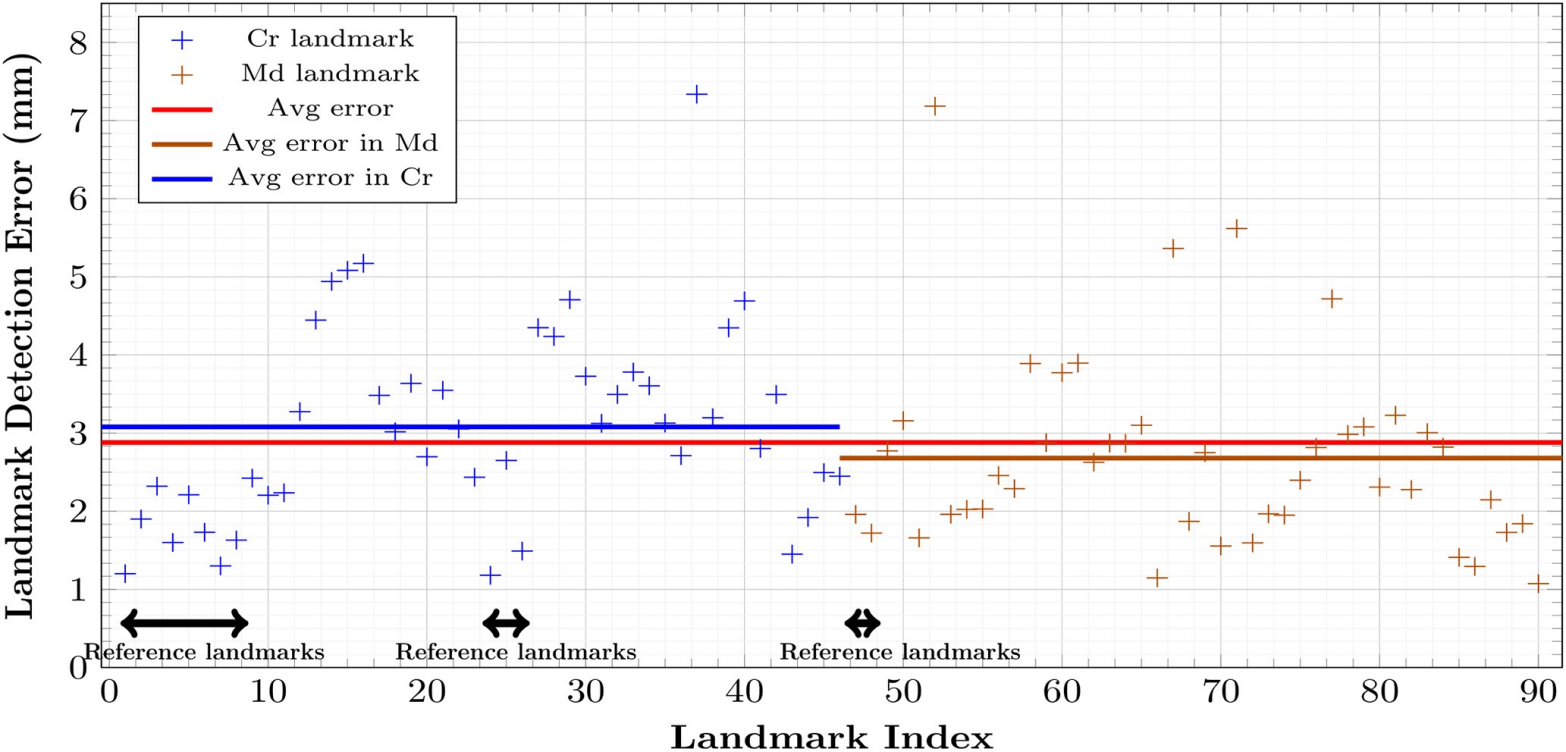
Final localization errors (mm) of 90 cephalometric landmarks after coarse-to-find detection over 9 test data. Blue and brown dots denote errors for cranial and mandibular landmarks, respectively. Red, blue, and brown lines represent average error over all, cranial, and mandibular landmarks.

The detection of the 10 reference landmarks ($R_{\#}$) provided very accurate and robust results (see Table 1 and Fig 6). These results almost meet clinical requirements, while the intra-observer repeatability has a precision of less than 1 mm and the overall median inter-observer precision is approximately 2 mm in the 3D landmarking system [22].

**Table 1:** Mean detection error for 10 reference landmarks. Most of the landmarks are annotated almost within clinical requirements.

Landmark	Mean (mm)	Landmark	Mean (mm)
**ANS**	1.2	**Na**	1.7
**Bregma**	1.9	**Or (R)**	1.3
**CFM**	2.32	**Po (R)**	1.63
**Or (L)**	1.6	**MF (L)**	1.96
**Po (L)**	2.21	**MF (R)**	1.72

By using reference landmarks, we normalized the landmark data via uniform scaling by fixing the cranial volume of each subject as the average value of the cranial volume for the training dataset.

### 3.3 Results of VAE

For the local-to-global detection, the VAE was trained using 45000 epochs, a full batch-size, and a learning rate of 0.001. Here, the full batch-size indicates that our dataset was not divided into several batches in the training process. These learning parameters were empirically chosen by comparing validation errors, which were obtained by varying parameters when training VAEs.

To investigate the effect on the dimension of the latent space, we trained VAE with varying the latent space size. The latent dimension is preferred to be as small as possible compared to that of the vector of reference landmarks ($R^{30}$) as well as global landmarks ($R^{270}$). Taking this into account, the latent dimension (9) and epochs (45000) were chosen as empirical optimal values based on the validation error. Table 2 shows the variation of the averaged test error for the epoch and latent space dimension. The error tendency for the test set was almost the same as that for the validation set.

**Table 2:** Mean detector error (mm) of VAE over 9 test data. This is obtained by varying the number of epochs and latent space dimension.

dim/epoch	35000	40000	45000	50000	55000
**3**	4.93	5.11	5.20	5.19	5.23
**5**	4.18	4.29	4.26	4.41	4.51
**7**	3.23	3.32	3.35	3.41	3.36
**9**	3.04	3.27	3.06	3.21	3.18
**11**	3.55	3.34	3.30	3.23	3.27
**13**	3.09	3.19	3.19	3.10	3.15
**15**	3.12	3.16	3.14	3.00	3.08

The averaged representation errors of VAE for 9 test data were 2.89 mm, 3.11 mm, and 3.06 mm for the cranial, mandibular, and all landmarks.

### 3.4 Results of the initial local-to-global detection

**Fig 7:**
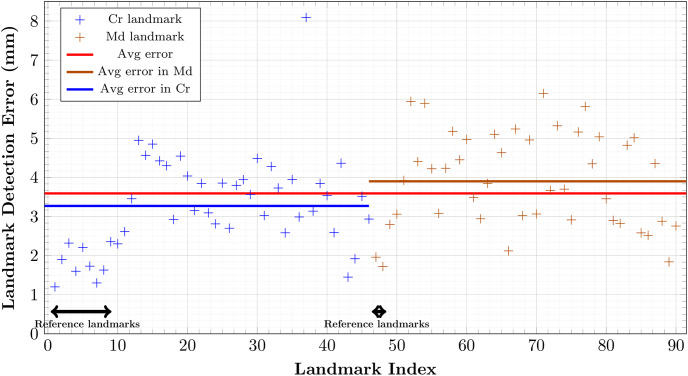
VAE-based local-to-global estimation errors (mm) of 90 landmarks over 9 test data. From 10 reference landmarks obtained in the first step, 90 landmarks are roughly estimated. Blue and brown dots denote errors for the cranial and mandibular landmarks, respectively. Red, blue, and brown lines represent average error over all, cranial, and mandibular landmarks.

The nonlinear map Φ was trained with 5400 epochs, a full batch-size, and a learning rate of 0.0001. For each landmark, Fig 7 shows the performance evaluation achieved using 9 test data with respect to the averaged error in the sense of (17). The mean detection error was 3.27 mm for the cranial landmarks, 3.90 mm for the mandibular landmarks, and 3.59 mm for all landmarks. The error of the cranial landmark estimation was much smaller than that of the mandibular landmark estimation.

The reference landmark detection outputs were selected as the estimation results instead of VAE outputs. This is because the 2D CNN is specially designed to detect the reference landmarks that are placed on a point with a morphologically distinct feature, whereas the VAE-based estimation focuses on capturing global landmark patterns within acceptable tolerance rather than on accurately detecting the specific landmarks. This also applied for the 2D CNN-based cranial landmark detection (in Section 2.3.3).

### 3.5 Result for coarse-to-fine detection

#### 3.5.1 Mandibular landmark detection

For fine detection of the mandibular landmarks, 3D image patches were extracted with size of 80 × 80 × 80 voxels (≈ 4 × 4 × 4 cm^3^). To generate the training data in (11), the center location of patch was varied to cover 2 times the maximum error of the initial estimation of $R_{◇}$ for the training data. Using the parameters of 20000 epochs, a full batch size, and a learning rate of 0.0005, nine 3D CNNs were trained.

**Fig 8:**
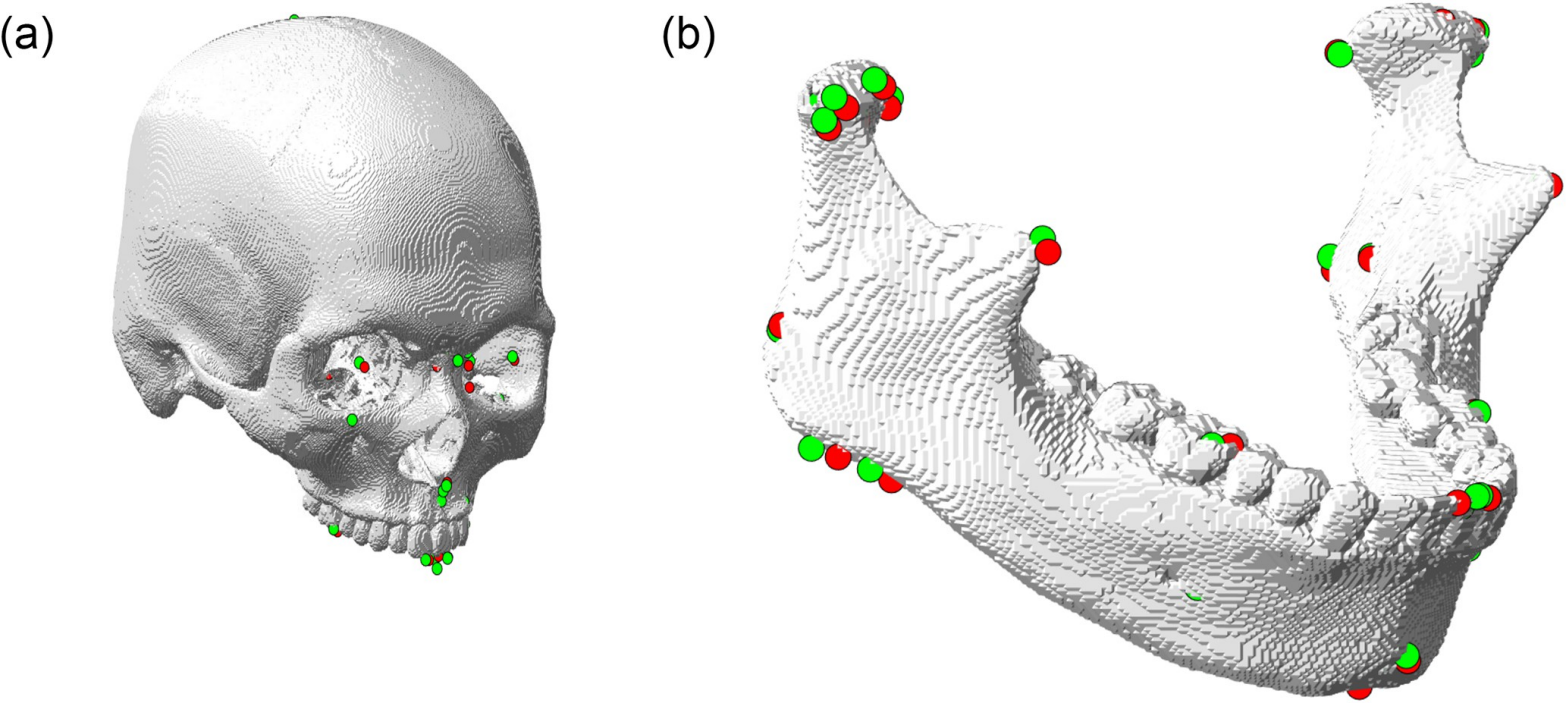
Qualitative evaluation of detection for (a) cranial landmarks and (b) mandibular landmarks. The red and green dots denote the ground truth and detected output landmarks respectively.

Figs 6 and 8(b) show the quantitative and qualitative results of the 3D CNNs. The mean distance error decreased to 2.68 mm from the initial detection error of 3.90 mm. The proposed method achieved an error range of 1 to 4 mm for the detection of most landmarks. In addition, as shown in Fig 10(b), the proposed method significantly reduced the mean and variance of error for the test subjects, compared to the initial detection.

#### 3.5.2 Cranial landmark detection

To generate the partially integrated image ***x***^mid^, we set the interval for the truncated volume as ± 7.5 mm *v*_1_-directionally from the midsagittal plane. Next, 2D image patches were cropped into sizes of 80 × 80 pixels (≈ 4 × 4 cm^2^). For training the 2D CNNs, we used the learning parameters of 5000 epochs, a full batch-size, and a learning rate of 0.0001.

**Fig 9:**
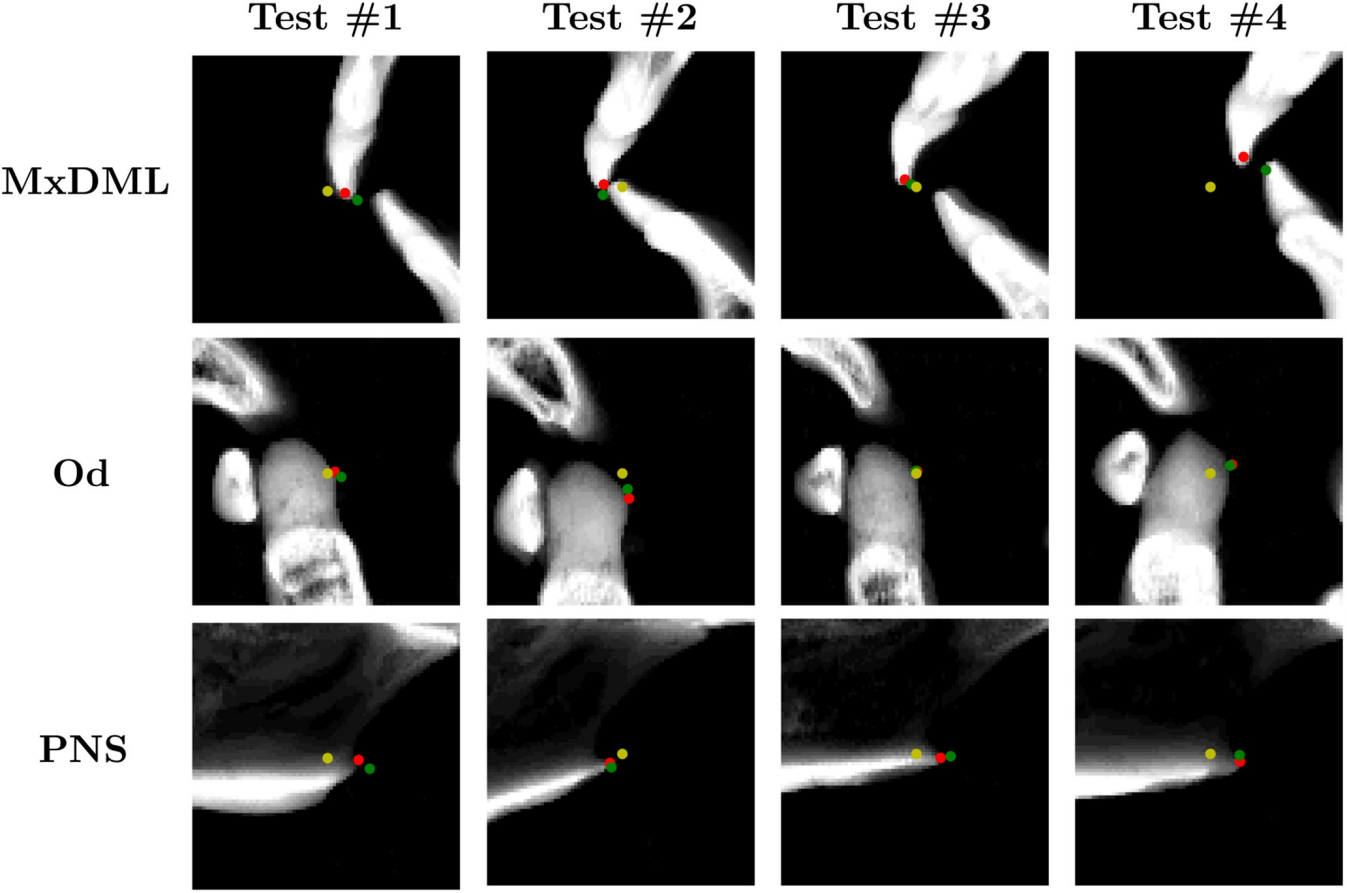
Results of coarse-to-fine landmark detection on 2D patch. Yellow dot is the output of the coarsely detected VAE. Green dot is the output of detection using patch-based CNN. Red dot is the ground truth.

In Fig 9 and Table 3, qualitative and quantitative evaluations of the 2D CNN-based detection of three cranial landmarks on the midsagittal plane are provided. The detection achieved relatively accurate annotation on the three target landmarks.

**Table 3:** Error evaluation of the landmarks on the midsagittal plane. Initial error and 2D CNN error are presented in the table. The errors are reduced after the 2D CNN is applied.

Landmark name	Error before 2D CNN (mm)	Error after 2D CNN (mm)
**MxDML**	2.81	1.18
**Od**	3.85	2.65
**PNS**	2.70	1.49

For the estimation of all cranial landmarks, VAE $\left(E^{\text{cr}},D^{\text{cr}}\right)$ was trained with 80000 epochs, a full batch, and a learning rate of 0.001. The map Φ^cr^ was trained with 23000 epochs, a full batch-size, and a learning rate of 0.0001. The latent dimension was empirically set to 15.

**Fig 10:**
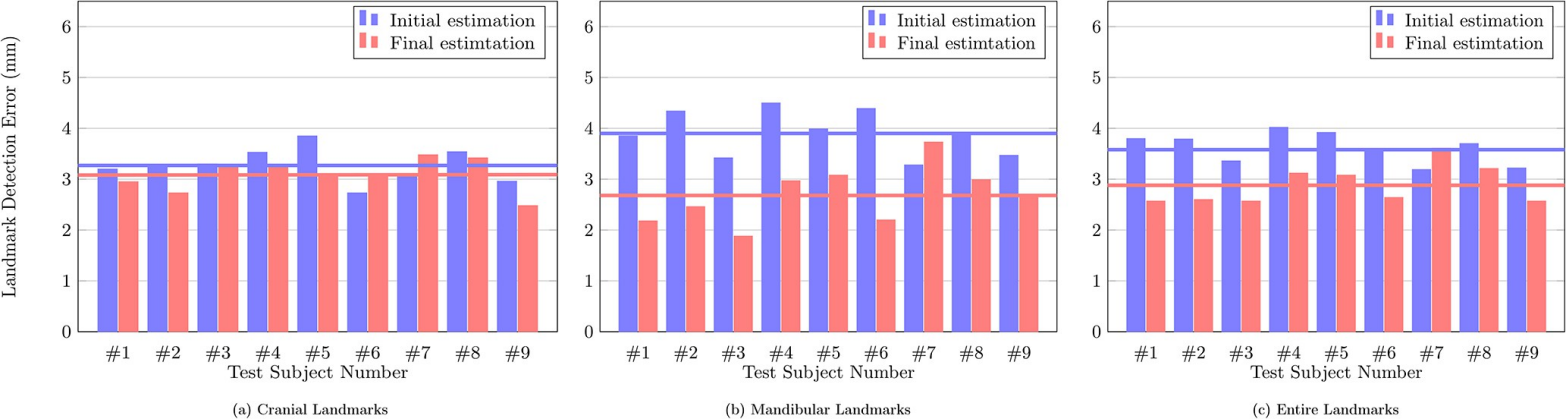
The mean detection error of the cranial, mandibular, and all landmarks for each of the 9 test data. Blue bars represent the error from the initial estimation, red bars represent the error from the final estimation, and the lines represent the averaged error over test subjects. (a) Cranial Landmarks. (b) Mandibular Landmarks. (c) Entire Landmarks.

Figs 6 and 8(a) show the final cranial landmark estimation results in quantitative and qualitative formats, respectively. The mean detection error for all cranial landmarks was 3.08 mm, decreasing from the initial estimation error of 3.27 mm (Fig 10(a)). The error for most cranial landmarks fell within the range of 1 to 4 mm.

In terms of all landmarks, as described in Fig 10(c), our proposed method achieved an error of 2.88 mm (Fig 6), which is much lower than the initial detection error of 3.59 mm (Fig 7).

## 4 Discussion and conclusion

This article proposes a fully automatic landmarking system for 3D cephalometry in 3D CT. The proposed method provides the accurate and reliable identification of cephalometric landmarks that can be used in subsequent clinical studies, such as in the development of morphometrical guidelines for diagnosis, surgical planning, and the treatment of craniofacial diseases. The proposed semi-supervised method is designed to use many anonymized landmark dataset to address the severe shortage of training CT data. Currently, only 24 CT data pairs are available due to the legal and ethical restrictions on medical data, while approximately 200 anonymized landmark data are available.

The proposed method is based on the benchmark model [13], which provides 3.63 mm error for annotation of 90 landmarks. This model motivated the backbone structure of the coarse estimation step. The proposed method reduces the average detection error from 3.63 mm to 2.88 mm by employing the coarse-to-fine detection, where appropriate strategies for mandibular and cranial landmarks were considered for their different properties. We expect the detection accuracy to be further improved with increasing amount of available training data.

While it may be possible to directly learn the map from the partial knowledge to the global landmarks, the use of VAE is an effective approach for obtaining a meaningful latent representation in terms of skull morphology while learning the local-to-global estimation map. Human skull morphology follows certain patterns and the positions of landmarks are closely interrelated. A previous study [13] provided empirical evidence that VAE can learn a low-dimensional representation that is strongly associated with the factors determining facial skeletal morphology. It is also a well-known advantage of VAE that the learned latent space is dense and smooth [13, 14, 23]. Hence, it is expected that the VAE-based local-to-global estimation map not only provides the connection between partial and global landmarks but also follows the merits of VAE for latent representation.

Landmarks on the cranium have smaller variability between subjects compared to those on mandible due to the rigid property of the cranium; therefore, cranial landmarks are relatively suitable for the effective estimation in the sense of finding certain common patterns over the training dataset. Moreover, the use of 3D CNN-based fine annotation for all landmarks requires high computational memory consumption and power budget due to the increased use of 3D networks. Hence, the VAE-based approach can be regarded as an effective strategy to finely detect cranial landmarks with a sufficient level of accuracy. Meanwhile, the positional estimation of the summit position of the cranium (SC) obtained from the relation learned via VAE exhibited the lowest accuracy (see Fig 6). This appears to have occurred because the SC may weakly depend on the positions of other landmarks. A rigorous factor analysis using VAE may be undertaken in future research.

The proposed method has the potential to alleviate the experts’ hectic workflow by introducing an automated cephalometric landmarking with high accuracy. In clinical practice, our method allows all 3D landmarks to be estimated from partial information obtained via 3D CT data. Although the error level of some landmarks does not meet the requirement of clinical applications (less than 2 mm), the proposed method may still aid in decisions of clinicians in determining landmark positions, thereby improving their working processes.

Recently, as concerns about the radiation doses have increased, there have been attempts to use dental cone-beam CT for cephalometric analysis instead of the conventional multi-detector CT because cone-beam CT utilizes a much lower radiation dose than multi-detector CT. The investigation of an automated 3D landmarking system for cone-beam CT will therefore be a topic of our future research.

## Supporting information

S1 Table About 90 cephalometric landmarks. (ZIP)

## References

[1] Tenti F., Cephalometric analysis as a tool for treatment planning and evaluation. The European Journal of Orthodontics. 1981;3(4): 241–245. doi: 10.1093/ejo/3.4.241 6945994

[2] Proffit W., Fields H., Larson B., and Sarver D. Contemporary Orthodontics Vol. 6th Edition. Mosby; 2018

[3] Pittayapat P., Limchaichana-Bolstad N., Willems G., and Jacobs R., Three-dimensional cephalometric anlaysis in orthodontics: a systematic review. Orthodontics & craniofacial research. 2014;17(2): 69–91. doi: 10.1111/ocr.12034 24373559

[4] Adams G. L., Gansky S. A., Miller A. J., Harrell W. E. Jr, and Hatcher D. C., Comparison between traditional 2-dimensional cephalometry and a 3-dimensional approach on human dry skulls. American journal of orthodontics and dentofacial orthopedics. 2004;126(4): 397–409. doi: 10.1016/j.ajodo.2004.03.023 15470343

[5] Nalcaci R., Öztürk F., and Sökücü O., A comparison of two-dimensional radiography and three-dimensional computed tomography in angular cephalometric measurements. Dentomaxillofacial Radiology. 2010;39(2): 100–106. doi: 10.1259/dmfr/82724776 20100922 PMC3520203

[6] Lee S.-H., Kil T.-J., Park K.-R., Kim B.C., Piao Z., and Corre P., Three-dimensional architectural and structural analysis-a transition in concept and design from Delaire’s cephalometric analysis. Int J Oral Maxillofac Surg. 2014;43: 1154–1160. doi: 10.1016/j.ijom.2014.03.012 24794759

[7] Arik S.Ö., Ibragimov B., and Xing L., Fully automated quantitative cephalometry using convolutional neural networks. J Med Imaging (Bellingham). 2017;4(1): 014501 doi: 10.1117/1.JMI.4.1.014501 28097213 PMC5220585

[8] Lindner C., Wang C.-W., Huang C.-T., Li C.-H., Chang S.-W., and Cootes T. F., Fully automatic system for accurate localisation and analysis of cephalometric landmarks in lateral cephalograms. Scientific reports. 2016;6: 33581 doi: 10.1038/srep33581 27645567 PMC5028843

[9] Codari M., Caffini M., Tartaglia G.M., Sforza C., and Baselli G., Computer-aided cephalometric landmark annotation for CBCT data. International journal of computer assisted radiology and surgery. 2017;12(1): 113–121 doi: 10.1007/s11548-016-1453-9 27358080

[10] Montufar J., Romero M., and Scougall-Vilchis R. J., Automatic 3-dimensional cephalometric landmarking based on active shape models in related projections. American Journal of Orthodontics and Dentofacial Orthopedics. 2018;153(3): 449–458 doi: 10.1016/j.ajodo.2017.06.028 29501121

[11] Lee S. M., Kim H. P., Jeon K., Lee S. H., and Seo J. K., Automatic 3D cephalometric annotation system using shadowed 2D image-based machine learning. Physics in medicine and biology. 2019;64(5): 055002. doi: 10.1088/1361-6560/ab00c9 30669128

[12] Kang S. H., Jeon K., Kim H., Seo J.K., and Lee S., Automatic three-dimensional cephalometric annotation system using three-dimensional convolutional neural networks: a developmental trial, Computer Methods in Biomechanics and Biomedical Engineering: Imaging & Visualization. 2020;8(2): 210–218.

[13] Yun H. S., Jang T.J., Lee S. M., and Seo J.K., Learning-based local-to-global landmark annotation for automatic 3d cephalometry. Physics in Medicine & Biology. 2020;65(8): 085018. doi: 10.1088/1361-6560/ab7a71 32101805

[14] Kingma D. P., and Welling M., auto-encoding variational bayes. arXiv preprint. 2013; arXiv:1312.6114.

[15] Vallabh R., Zhang J., Fernandez J., Dimitroulis G., and Ackland D. C., The morphology of the human mandible: A computational modelling study. Biomechanics and Modeling in Mechanobiology. 2019:1–16. 30826909 10.1007/s10237-019-01133-5

[16] Jang T. J., Kim K. C., Cho H. C., and Seo J. K., A fully automated method for 3d individual tooth identification and segmentation in dental CBCT. IEEE Transactions on Pattern Analysis and Machine Intelligence. 2022. 34077356 10.1109/TPAMI.2021.3086072

[17] Kyriakou Y., Meyer E., Prell D., and Kachelrieb M., Empirical beam hardening correction (EBHC) for CT. Medical physics. 2010;37(10): 5179–5187. doi: 10.1118/1.3477088 21089751

[18] Simonyan K., and Zisserman A., Very deep convolutional networks for large-scale image recognition. arXiv preprint. 2014:arXiv:1409.1556.

[19] Samet H., and Tamminen M., Efficient component labeling of images of arbitrary dimension represented by linear bintrees. IEEE transactions on pattern analysis and machine intelligence. 1988;10(4): 579–586. doi: 10.1109/34.3918

[20] Paszke A., Gross S., Massa F., Lerer A., Bradbury J., and Chanan G. et al., Pytorch: An imperative style, high-performance deep learning library. Advances in neural information processing systems. 2019:8026–2037.

[21] Kingma D. P., and Ba J., Adam: A method for stochastic optimization. arXiv preprint. 2014; arXiv:1412.6980.

[22] Pittayapat P., Jacobs R., Bornstein M. M., Odri G. A., Kwon M. S., Lambrichts I., et al, A new mandible-specific landmark reference system for three-dimensional cephalometry using cone-beam computed tomography. European journal of orthodontics. 2016;38(6): 563–568. doi: 10.1093/ejo/cjv088 26683131

[23] Seo J. K., Kim K. C., Jargal A., Lee K., and Harrach B., A learning-based method for solving ill-posed nonlinear inverse problems: a simulation study of lung EIT. SIAM journal on Imaging Sciences. 2019;12(3): 1275–1295. doi: 10.1137/18M1222600

