# Supplementary material for: A semi-supervised learning approach for automated 3D cephalometric landmark identification using computed tomography
Source: PLoS One. 2022 Sep 28;17(9):e0275114. doi: 10.1371/journal.pone.0275114 (PMC9518928; doi:10.1371/journal.pone.0275114)
Supplement: S1 Table — (ZIP) [file pone.0275114.s001.zip › final_Supporting_information.pdf]

Supporting information

Table S1. About 90 cephalometric landmarks

Location	Index	Landmark	Description
cranium	1	ANS	Anterior nasal spine (reference)
cranium	2	Bregma	Bregma (reference)
cranium	3	CFM	Center of foramen magnum (reference)
cranium	4	Or (L)	Left orbitale (reference)
cranium	5	Po (L)	Left porion (reference)
cranium	6	Na	Nasion (reference)
cranium	7	Or (R)	Right orbitale (reference)
cranium	8	Po (R)	Right porion (reference)
cranium	9	♯16 tip	The mesiobuccal cusp tip of maxillary right first molar
cranium	10	♯26 tip	The mesiobuccal cusp tip of maxillary left first molar
cranium	11	ANS'	Constructed ANS point
cranium	12	AO	Anterior occlusal point
cranium	13	FC	Falx cerebri
cranium	14	Clp (L)	Left posterior clinoid process
cranium	15	EC (L)	Left eyeball center
cranium	16	FM (L)	Left frontomaxillary suture
cranium	17	Hyp (L)	Left hypomochlion
cranium	18	M (L)	Left junction of nasofrontal, maxillofrontal, and maxillonasal sutures
cranium	19	NP (L)	Left nasopalatine foramen
cranium	20	Pti (L)	Left inferior pterygoid point
cranium	21	Pts (L)	Left superior pterygoid point
cranium	22	U1 apex (L)	Left upper incisal apex
cranium	23	U1 tip (L)	Left upper incisal tip
cranium	24	MxDML	Maxillary dental midline
cranium	25	Od	Odontoid process
cranium	26	PNS	Posterior nasal spine
cranium	27	Clp (R)	Right posterior clinoid process
cranium	28	EC (R)	Right eyeball center
cranium	29	FM (R)	Right frontomaxillary suture
cranium	30	Hyp (R)	Right hypomochlion
cranium	31	M (R)	Right junction of nasofrontal, maxillofrontal, and maxillonasal sutures
cranium	32	Np (R)	Right nasopalatine foramen
cranium	33	Pti (R)	Right inferior pterygoid point
cranium	34	Pts (R)	Right superior pterygoid point
cranium	35	U1 apex (R)	Right upper incisal apex
cranium	36	U1 tip (R)	Right upper incisal tip
cranium	37	SC	Summit of cranium
cranium	38	mid-Clp	Midpoint between right and left posterior clinoid point
cranium	39	mid-EC	Midpoint between EC (L) and EC (R)
cranium	40	mid-FM	Midpoint between FM (L) and FM (R)
cranium	41	mid-M	Midpoint between M (L) and M (R)
cranium	42	mid-Np	Midpoint between Np (L) and Np (R)
cranium	43	mid-Or	Midpoint between Or (L) and Or (R)
cranium	44	mid-Po	Midpoint between Po (L) and Po (R)
cranium	45	mid-Pti	Midpoint between Pti (L) and Pti (R)
cranium	46	mid-U1 tip	Midpoint between U1 tip (L) and U1 tip (R)
mandible	47	MF (L)	Left mental foramen (reference)
mandible	48	MF (R)	Right mental foramen (reference)
mandible	49	♯36 tip	The mesiobuccal cusp tip of mandibular left first molar
mandible	50	♯46 tip	The mesiobuccal cusp tip of mandibular right first molar
mandible	51	CON (L)	Left condylar point
mandible	52	COR (L)	Left coronoid point
mandible	53	Cp (L)	Left posterior condylar point
mandible	54	Ct-in (L)	Left medial temporal condylar point
mandible	55	Ct-mid (L)	Midpoint between left Ct-in and Ct-out
mandible	56	Ct-out (L)	Left lateral temporal condylar point

mandible	57	F (L)	Left mandibular foramen
mandible	58	Go-in (L)	Left inferior gonion point
mandible	59	Go-mid (L)	Midpoint between left posterior and inferior gonion point
mandible	60	Go-post (L)	Left posterior gonion point
mandible	61	L1 apex (L)	Root apex of left mandibular central incisor
mandible	62	L1 tip (L)	Incial tip midpoint of left mandibular central incisor
mandible	63	LCP (L)	Left lateral condylar point
mandible	64	MCP (L)	Left medial condylar point
mandible	65	a-Go notch (L)	Left antegonial notch
mandible	66	mid-F MF (L)	Midpoint between left mandibular foramen and mental foramen
mandible	67	Me (anat)	Anatomical menton
mandible	68	MnDML	Mandibular dental midline
mandible	69	Pog	Pogonion
mandible	70	CON (R)	Right condylar point
mandible	71	COR (R)	Right coronoid point
mandible	72	Cp (R)	Right posterior condylar point
mandible	73	Ct-in (R)	Right medial temporal condylar point
mandible	74	Ct-mid (R)	Midpoint between right Ct-in and Ct-out
mandible	75	Ct-out (R)	Right lateral temporal condylar point
mandible	76	F (R)	Right mandibular foramen
mandible	77	Go-in (R)	Right inferior gonion point
mandible	78	Go-mid (R)	Midpoint between right posterior and inferior gonion point
mandible	79	Go-post (R)	Right posterior gonion point
mandible	80	L1 apex (R)	Root apex of right mandibular central incisor
mandible	81	L1 tip (R)	Incial tip midpoint of right mandibular central incisor
mandible	82	LCP (R)	Right lateral condylar point
mandible	83	MCP (R)	Right medial condylar point
mandible	84	a-Go notch (R)	Right antegonial notch
mandible	85	mid-F MF (R)	Midpoint between right mandibular foramen and mental foramen
mandible	86	mid-Cp	Midpoint between right and left posterior condylar point
mandible	87	mid-F	Midpoint between F (L) and F (R)
mandible	88	mid-L1 tip	Midpoint between L1 tip (L) and L1 tip (R)
mandible	89	mid-MF	Midpoint between MF (L) and MF (R)
mandible	90	midpoint of mid-F MF (R/L)	Midpoint between mid-F MF (R) and mid-F MF (L)
